# The Efficacy of Psychological Intervention on Body Image in Breast Cancer Patients and Survivors: A Systematic-Review and Meta-Analysis

**DOI:** 10.3389/fpsyg.2021.611954

**Published:** 2021-03-01

**Authors:** Valeria Sebri, Ilaria Durosini, Stefano Triberti, Gabriella Pravettoni

**Affiliations:** ^1^Department of Oncology and Hemato-Oncology, University of Milan, Milan, Italy; ^2^Applied Research Division for Cognitive and Psychological Science, IEO, European Institute of Oncology IRCCS, Milan, Italy

**Keywords:** breast cancer, body image, sexual functioning, psychological interventions, cancer survivors

## Abstract

The experience of breast cancer and related treatments has notable effects on women's mental health. Among them, the subjective perception of the body or body image (BI) is altered. Such alterations deserve to be properly treated because they augment the risk for depression and mood disorders, and impair intimate relationships. A number of studies revealed that focused psychological interventions are effective in reducing BI issues related to breast cancer. However, findings are inconsistent regarding the dimension of such effects. This meta-analysis synthesizes and quantifies the efficacy of psychological interventions for BI in breast cancer patients and survivors. Additionally, since sexual functioning emerged as a relevant aspect in the BI distortions, we explored the efficacy of psychological interventions on sexual functioning related to BI in breast cancer patients and survivors. The literature search for relevant contributions was carried out in March 2020 through the following electronic databases: Scopus, PsycINFO, and ProQUEST. Only articles available in English and that featured psychological interventions for body image in breast cancer patients or survivors with controls were included. Seven articles with 17 dependent effect sizes were selected for this meta-analysis. Variables were grouped into: *Body Image* (six studies, nine dependent effect sizes) and *Sexual Functioning Related to the Body Image* in breast cancer patients and survivors (four studies, eight dependent effect sizes). The three-level meta-analysis showed a statistically significant effect for *Body Image* [*g* = 0.50; 95% CI (0.08; 0.93); *p* < 0.05] but no significant results for *Sexual Functioning Related to Body Image* [*g* = 0.33; 95% CI (−0.20; 0.85); *p* = 0.19]. These results suggest that psychological interventions are effective in reducing body image issues but not in reducing sexual functioning issues related to body image in breast cancer patients and survivors. Future review efforts may include gray literature and qualitative studies to better understand body image and sexual functioning issues in breast cancer patients. Also, high-quality studies are needed to inform future meta-analyses.

## Introduction

Breast cancer is one of the most common tumors among women (Ferlay et al., 2015; Andreis et al., 2018). Standard intervention approach includes surgery, adjuvant therapies, chemotherapy, hormonotherapy, and radiotherapy (Early Breast Cancer Trialists' Collaborative Group, 2011; Serletti et al., 2011). Despite a fairly good prognosis, cancer diagnosis and treatments concur to both negative physical and psychological long term-side effects that affect patients' and survivors' quality of life (QOL; Sterba et al., 2014; Ahmad et al., 2015; Williams and Jeanetta, 2016). Undesirable appearance-related side effects [i.e., loss or deformities in the breast(s), visible scarring, hair loss, skin discoloration, alopecia, muscle weakness, and weight fluctuation] alter the perception of Body Image (BI) and develop intensive negative feelings (Gorini et al., 2015; Fioretti et al., 2017; Yang et al., 2017).

BI is generally defined as “internal representation of one's own outer appearance” (Thompson et al., 1999, p. 4) that involves the mental representation of one's own body and related emotions within an overall sense of bodily self (Lewis-Smith et al., 2018; Sebri et al., 2020b). Perceptual (i.e., accuracy of estimated body size), attitudinal (i.e., subjective satisfaction about one's own body), cognitive (i.e., involvement in appearance belief about the body), affective (i.e., sensations and emotions), and behavioral dimensions (i.e., compensatory behaviors such as dieting and physical activity) are involved (Cash and Smolak, 2011). BI construct is indeed relevant because of impacts on the quality of life (Torres et al., 2020). Consequently, breast cancer patients and survivors' evaluation of their own physical appearance as well as attitudes toward the body notably change, especially in terms of femininity and sexuality (Maass et al., 2015; Sherman et al., 2018). Mastectomy surgery or conservation, for example, may threaten overall self-satisfaction and evoke multiple changes in body perception mediated by sensations within breast and chest never experienced before (Falbjork et al., 2013; Paterson et al., 2015). BI issues are strictly related to sexual functioning in breast cancer survivors. Sexual dysfunctions (i.e., sexual arousal, dyspareunia, fatigue, and loss or decrease in sexual desire and pleasure) occur frequently even beyond the acute phase of treatments, leading to dissatisfaction that becomes one of the most problematic aspects of survivors' life (Emilee et al., 2010; Male et al., 2016). Patients' and survivors' BI in terms of sexuality is altered by the fear of loss of fertility, induced menopause, and perception of sexual unattractiveness, linked also to perceived partner's greater difficulties to understand their feelings (Woertman and Van den Brink, 2012).

The subjective changed experience within one's own BI and related emotions impact on patients' and survivors' social relationships too (Dua et al., 2015; Triberti et al., 2019b). In general, women constantly self-scrutinize themselves compared to cultural stereotypes of physical appearance (Triberti et al., 2017a,b). Following the Self Discrepancy Theory by Higgins (1987), the discrepancy between one's own current and desired self-representations leads to feelings of dissatisfaction and emotional distress and promotes habitual self-surveillance and evaluation, according to theories of self-objectification (Fredrickson and Roberts, 1997). As a result, patients and survivors severely worry about physical appearance and develop the belief that others observe and evaluate their bodies continually (Hunter, 2015). In this regard, the construction of femininity and sexuality after illness depends not only on patients' and survivors' post-treatment experiences but also on the intrapsychic negotiation within their social and relational context (Male et al., 2016).

To sum up, a growing body of literature evidences that the experience of breast cancer seriously infringes patients' and survivors' BI and the general sense of self (Boquiren et al., 2013; Jabłoński et al., 2019) until years after diagnosis and treatments (Falk Dahl et al., 2010). For this reason, several psychological interventions have been proposed that address breast cancer survivors' BI issues and sexual functioning (Park et al., 2015). Most of these are programs based on cognitive-behavioral/existential, educational, supportive emotionally expressive, interpersonal, and psychosocial approaches (Blanco et al., 2014). Their main objectives are to decrease psychological distress providing problem-solving methods and relaxation and to modify the perception of one's own body. Furthermore, literature shows the efficacy of novel and mixed approaches including psychotherapy-based interventions, physical exercise, and art/dance therapy to improve well-being in cancer patients and survivors (Björneklett et al., 2013), as well as cosmetic educational programs to promote self-esteem (Park et al., 2015). For example, mixed interventions featuring psychological support and physical activity (aerobic and resistance training especially) promote personal strength and quality of life (Fong et al., 2012; Benton et al., 2014) as well as improvements in cognitive abilities such as attention, working memory, and decision making (Sebri et al., 2019, 2020a). Regarding type of session, structured group, couple intervention, and formal one-to-one psychotherapy are the main formats of support within clinical settings (Fingeret et al., 2014). Among different methodological approaches, Cognitive Behavioral Therapy (CBT) has been found particularly promising as a time-limited and goal-oriented psychotherapeutic approach (Fingeret et al., 2014).

With the aim of evaluating the usefulness of different programs and approaches, Lewis-Smith et al. (2018) highlighted that breast cancer patients and survivors perceive psychological interventions as acceptable, feasible, and effective to address BI issues (McLean et al., 2011). Nevertheless, evaluations of psychological interventions' long-term impact are limited due to a general lack of methodological rigor across studies (Lewis-Smith et al., 2016, 2018). Most psychological interventions do not adopt an exclusive focus on BI, which tends to be addressed as a small component of larger interventions. Also, there is no clear, unified information about the dimension of such positive effects in the literature. For this reason, a meta-analytic approach to the issue is in order.

The aim of this study is to evaluate the efficacy of different kinds of psychological interventions on BI and sexual functioning in breast cancer patients and breast cancer survivors. Specifically, this study assessed which typology of psychological intervention is the most effective to improve BI in breast cancer patients and survivors. Based on the existing literature, we expected that psychological interventions would promote benefits on BI and sexual functioning related to BI in cancer patients and survivors.

## Method

The literature search for relevant contributions was carried out in March 2020 through an electronic computer-based search on the following databases: Scopus, PsycINFO, and ProQUEST. The PICOS model was used as a tool for developing search strategies for this meta-analysis and eligibility criteria. This model includes the patient or problem (P), the intervention or exposure (I), the comparison intervention or exposure (C), and the clinical outcome of interest (O) (Eriksen and Frandsen, 2018), and (S) Study type. Following this model, records were searched using “psychological intervention” OR “psychological therapy” OR “psychological support” OR “psychotherapy” AND “body esteem” OR “bodily self” OR “body image” OR “body consciousness” AND “breast cancer” as key terms in the title or in the abstract of the manuscripts. Only articles available in English were included. The authors did place a *priori* restrictions by excluding “gray literature” such as conference abstracts, other non-peer-reviewed sources, and doctoral dissertation in the attempt to improve review manageability (Beatty et al., 2018). No other limitations were placed in reference to age of participants, statistical presentation of results, time period of publications, or study type. Studies were included in meta-analysis if they met the following criteria: (1) studies that examined the efficacy of psychological interventions for BI on cancer patients or survivors compared with a control group; (2) studies that included a measure of BI that was consistent with any dimension of BI (e.g., subjective evaluation and/or perceptual); (3) between-group outcome data of mixed-method design studies or between-group research studies. If different times were analyzed in the study, we considered only the last outcome point available; (4) studies written in English. Previous studies showed that there was nearly no evidence of a systematic bias from English language restriction in meta-analyses (e.g., Morrison et al., 2012; McKenzie et al., 2019; see Table 1 for a detailed description of the search strategy and eligibility criteria).

**Table 1:** The search strategy.

**Selection criteria**		**Keywords**	**Inclusion criteria**
Participants	#1	“breast cancer” (title and abstract)	We included studies with breast cancer patients or survivors
Interventions	#2	“psychological intervention” (title and abstract) OR “psychological therapy” (title and abstract) OR “psychological support” (title and abstract) OR “psychotherapy” (title and abstract)	We included studies that examined the efficacy of psychological interventions for body image (BI) on cancer patients or survivors compared with a control group. We included only intervention that was conducted by trained psychologists or studies in which the psychological intervention is used alone, and not administered in combination with other educational techniques (which makes it difficult to understand the effectiveness of the psychological interventions)
The comparison intervention or exposure	#3		We included studies in which participants in the control group participated in an active or non-active intervention
Outcome	#4	“body esteem” (title and abstract) OR “bodily self” (title and abstract) OR “body image” (title and abstract) OR “body consciousness” (title and abstract)	We included studies that assess a measure of BI that was consistent with any dimension of BI (e.g., subjective evaluation and/or perceptual). We included between-group outcome data of mixed-method designs studies or between-groups research studies. If different times were analyzed in the study, we considered only the last outcome point available
Study type	#5	No restriction	No restriction
Search combination		#1 AND #2 AND #3 AND #4	
**Database search**			
Language			We included studies written in English
Electronic databases		Scopus, PsycINFO, and ProQUEST	

The effect sizes (Hedges' *g*) were reported or computed based on the information provided in the article. If an article did not provide appropriate statistics to compute effect sizes, it was excluded from this meta-analysis. Of note, since we only aimed to explore the efficacy of psychological interventions for BI in breast cancer patients and survivors, we decided to exclude studies that did not use psychological interventions, studies that explicitly state in the article that the intervention was not conducted by trained psychologists, or studies in which the psychological intervention is not used alone, but was administered in combination with other educational techniques (which makes it difficult to understand the effectiveness of the psychological interventions). This meta-analysis has been registered with the International Prospective Register for Systematic Reviews with ID number CRD42020203021 (available from https://www.crd.york.ac.uk/prospero/display_record.php?ID=CRD42020203021).

### Coding of Studies and Data Extraction

During the first screening stage, two researchers (VS and ID) independently coded the studies. After removing duplicates, the titles and abstracts of 180 articles (20% of 903 articles potentially relevant for inclusion) were independently screened based on the inclusion criteria, in order to exclude irrelevant studies for this meta-analysis. Inter-rater agreement coefficient (Cohen's *k;* McHugh, 2012) was equal to 0.91. Discrepancies between the raters were resolved by referring back to the original article and through discussions with the third author (ST) to reach a consensus. In the next stage, 20% of 32 full-text articles assessed for eligibility (six articles) were independently screened by researchers to assess their relevance. Inter-rater agreement coefficient (Cohen's *k*) was equal to 1.00.

For each of the selected study, two researchers extracted in a blinded manner (1) the basic information (e.g., authors, publication year), (2) the type of psychological intervention, (3) the type of control group (alternative intervention vs. no intervention), (4) the format of the intervention (individuals, groups, couples), (5) the sample size and sample characteristics [i.e., participants (patients or survivors); mean age of participants], and (6) the instruments used in the study and the variables explored. Inter-rater reliability analysis revealed a perfect agreement between researchers was reached.

### Assessment of Study Quality

For what regard the studies included in the meta-analysis, the assessment of study quality was conducted by the first two authors of this manuscript independently. Discrepancies were resolved through discussions with the third author. We adapted the eight-criteria defined by Cuijpers et al. (2010) and already used in other meta-analyses for assessing study quality (David et al., 2018; Hoppen and Morina, 2020). We also included three additional items to further explore the quality of the studies included in this meta-analysis (see Table 2).

**Table 2:** Criteria of the assessment of study quality.

**In Cuijpers et al. (2010, p. 212, 213)**	**In this meta-analysis**
(1) “Participants met diagnostic criteria for a depressive disorder (as assessed with a personal diagnostic interview, such as CIDI, SCID, or SADS, and using a diagnostic system such as DSM or Research Diagnostic Criteria)”	(1) Breast cancer patients or breast cancer survivors
(2) “The study referred to the use of a treatment manual (either a published manual, or a manual specifically designed for the study)”	(2) Detailed description of the psychological approach, timing, procedure, and sessions
(3) “The therapists who conducted the therapy were trained for the specific therapy, either specifically for that study or as a general training”	(3) The psychologists who conducted the psychological treatment were trained for the specific psychological intervention
(4) “Treatment integrity was checked during the study (by supervision of the therapists during treatment or by recording of treatment sessions or by systematic screening of protocol adherence by a standardized measurement instrument)”	(4) “Treatment integrity was checked during the study (by supervision of the psychologists during treatment or by recording of treatment sessions or by systematic screening of protocol adherence by a standardized measurement instrument)”
(5) “Data were analyzed with intention-to-treat analyses, in which all persons who were randomized to the treatment and control conditions initially were included in the analyses”	(5) Same
(6) “The study had a minimal level of statistical power to find significant effects of the treatment, and included ≥ 50 persons in the comparison between treatment and control groups [this allows the study to find standardized effect sizes of d = 0.80 and larger, assuming a statistical power of 0.80 and α = 0.05; calculations in Stata (Stata Corp., USA)]”	(6) Not included
(7) “The study reported that randomization was conducted by an independent (third) party (this variable was positive if an independent person did the randomization, when a computer program was used to assign patients and survivors to conditions, or when sealed envelopes were used)”	(7) Same
(8) “Assessors of outcome were blinded and did not know to which condition the respondents were assigned to (this was only coded when the effect sizes were based on interviewer-based depression ratings; when only self-reports were used, it was assumed that this criterion was met)”	(8) Same
	**Additional items to further explore the quality of studies**
	(9) The control groups do not receive an intervention^*^
	(10) The absence of differential attritions between intervention and control groups (e.g., a great number of participants dropped out of the research study)^*^
	(11) The absence of reporting bias in the results^*^

* *Items included in this meta-analysis to further explore the quality of studies*.

### Quality Appraisal of the Studies

Independent assessments of the methodological quality of each study were conducted by three researchers (VS, ID, and ST) using the Cochrane risk of bias tool, version 2 (RoB 2; Higgins et al., 2011). The RbB 2 is based on some domains related to the quality appraisal of the studies and their biases (Sterne et al., 2019). For each study, the results of the risk of bias were differentiated as “low risk,” “some concerns,” and “high risk” by assessing each domain and its related specific risks. The overall risk of the study is considered low if the risk assessment of all the domains resulted in low (Higgins et al., 2011). Discrepancies in the quality of the studies were resolved through discussions between researchers.

### Data Analysis

Data analyses were conducted via the software *R* (the *rma.mv* function of the metafor package; Viechtbauer and Viechtbauer, 2015) and the *SPSS Statistical Software 20.0*.

We conducted six major sets of analyses:

1. First of all, an outlier analysis was conducted on the effect sizes included in this meta-analysis. An outlying effect was present when standardized *z* values exceeded +3.29 (Tabachnick and Fidell, 2013);
2. Hedges' *g* was computed for each comparison between the experimental and control groups using means and standard deviations. Hedges' *g* was interpreted as small (≤ 0.20), medium (=0.50), or large (≥0.80) (Cohen, 1988; Chalmers et al., 2014). Positive values indicate that the outcome is higher in breast cancer patients or survivors who received psychological interventions compared to participants who did not receive a psychological intervention;
3. Meta-analyses were run in order to assess the efficacy of psychological interventions on *Body Image* (Outcome 1) and *Sexual Functioning Related to Body Image* (Outcome 2) in breast cancer patients and survivors. In traditional meta-analysis, an important assumption is the independence between the included effect sizes (Rosenthal and Rubin, 1986; Assink and Wibbelink, 2016). This allows avoidance of overconfidence in the results and “inflated” estimates (Van den Noortgate et al., 2013; Assink and Wibbelink, 2016). Since this important precondition is violated in this meta-analysis, we used a three-level analysis (Assink and Wibbelink, 2016; Assink et al., 2018; van der Put et al., 2018; van der Put et al., 2020);
4. To deal with dependency of effect sizes (Van den Noortgate et al., 2013; Cheung, 2014), three levels of the model were included in the analysis:
  a) *Level 1 variance*: It refers to the sampling variance of the individual effect sizes;
  b) *Level 2 variance*: It refers to the variance between effect sizes from the same study;
  c) *Level 3 variance*: It refers to the differences between studies.
  (*Level 2* and *Level 3* variances are included as random terms in the model);
5. Publication bias was explored by inspecting the funnel plot graphs' asymmetry (Sterne and Egger, 2001) conducted with the trim-and-fill method and .rma function. If publication bias is absent, the funnel plot should assume a symmetric funnel shape. We also assessed whether small effects were underrepresented and whether large effects were missing (a great number of “missing” effect sizes suggested a bias to a greater extent; Assink et al., 2018) observing the white dots in the funnel plot (Higgins and Thompson, 2002).
6. Lastly, the presence or absence of heterogeneity across studies was tested by the *Q* statistic (Hedges and Olkin, 1985). The extent of such heterogeneity across studies was assessed using the *I*^2^ index (Higgins and Thompson, 2002). *I*^2^ index of 25, 50, and 75% indicate low, moderate, and high heterogeneity, respectively (Higgins et al., 2003). We also examined how the total variance was distributed over the three levels (*Level 1, Level 2*, and *Level 3*; Cheung 2014, formula 14, p. 2015). In order to determine whether the variance in *Level 2* and in *Level 3* were significant, we performed two separate one-tailed log-likelihood-ratio-tests. In these analyses the outcome of the full model was compared to the outcome of a model excluding one of the variance parameters. The model parameters were estimated using the restricted maximum likelihood estimation method. Lastly, we computed a prediction interval analysis (e.g., IntHout et al., 2016) in order to explore an approximate 95% range of underlying effects.

## Results

### Study Selection

**Figure 1:**
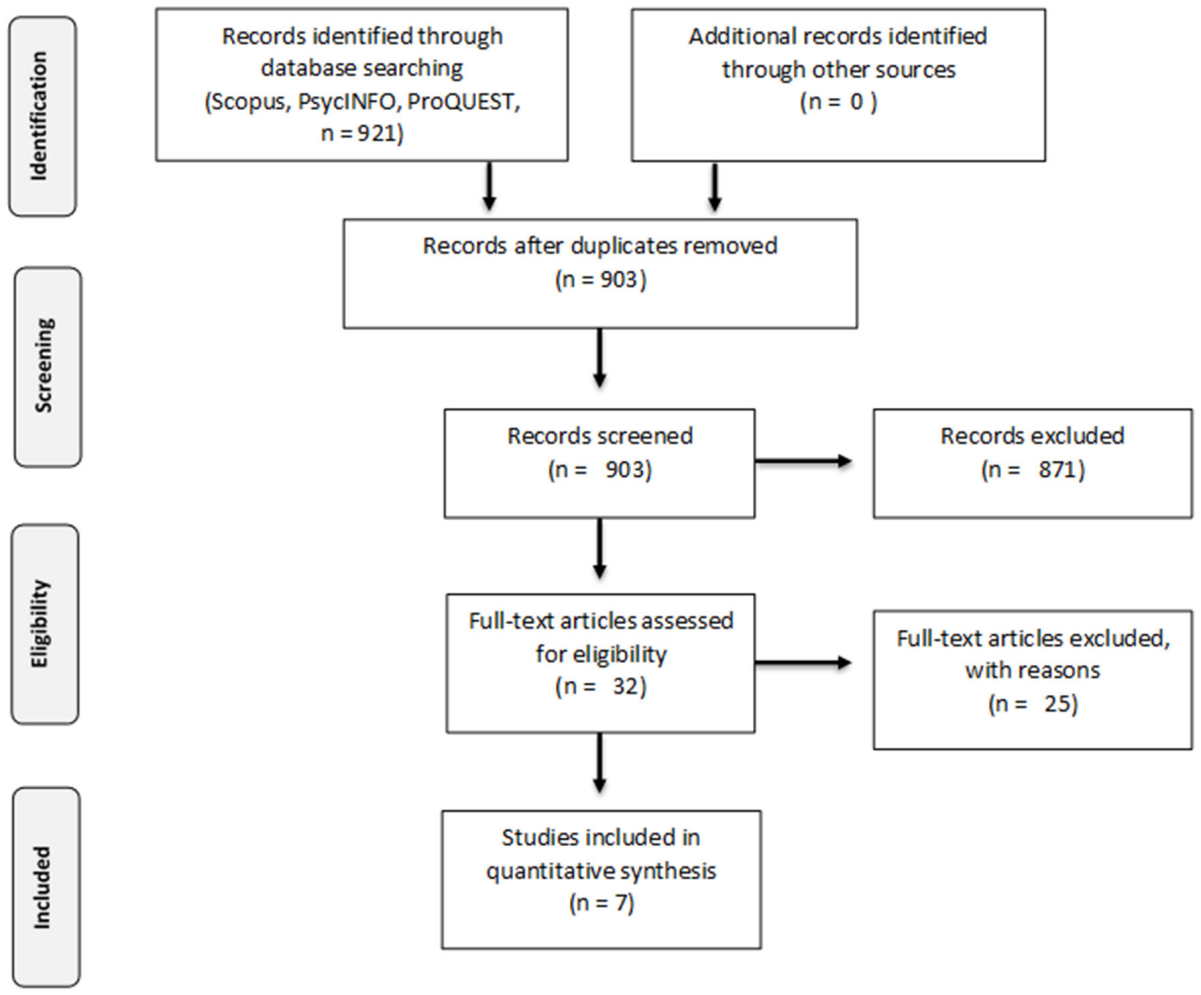
PRISMA flow diagram (Moher et al., 2009).

The meta-analysis was conducted following the guidelines and checklist of the Preferred Reporting Items for Systematic Reviews and Meta-Analyses (PRISMA; Liberati et al., 2009; Moher et al., 2009; Figure 1). Table 3 summarizes the Participants, Interventions, Control group intervention, and Outcome measures included in this meta-analysis. The initial search process returned 921 potentially relevant articles. After removing duplicates, 903 studies remained. Two researchers reviewed and screened these studies by reading the title and abstract, and based on the inclusion criteria, 32 were full-text screened. This selection procedure yielded a subset of seven usable empirical studies (Table 4). These studies were published between 2008 and 2014 and included a total sample size of 366 participants and 17 individual effect sizes. Each variable assessed in the selected studies was carefully analyzed by the authors and classified in the *Body Image* outcome (Outcome 1). Since the BI issues are strictly related to sexual functioning in breast cancer patients and survivors and sexual dysfunctions occur frequently even beyond the acute phase of treatments, we decided to explore also the effect of psychological interventions on *Sexual Functioning Related to Body Image* in breast cancer patients and survivors (Outcome 2).

**Table 3:** Description of the studies included in the meta-analysis following the PICOS model, The PICOS measure.

**Participants**	**Breast cancer patients or survivors**
Interventions	Psychological Interventions (Art therapy, psychosexual intervention, mindfulness-based stress reduction treatment, or metacognition group therapy, sexual life reframing program focused on the physical. psychological, and relational aspects of sexual health elements, psychosocial intervention program, mindfulness and dance/movement activities, web-based psychological intervention)
The comparison intervention or exposure	Non-active intervention (no intervention) vs. active intervention (usual care or expressive writing)
Outcome	*Body Image* (Outcome 1) or *Sexual Functioning related to Body Image* (Outcome 2)
Study type	Randomized and non-randomized control trials

**Table 4:** Detailed description of the studies included in the meta-analysis.

**Study**	**Population**	**Psychological intervention type**	**Control group**	**Intervention format**	***N (N* experimental group; *N* control group)**	**M age**
Svensk et al. (2009)	Patients	Art therapy (1 h/a week per 5 weeks, for a total of five sessions)	No intervention	Individual	41 (20;21)	59.5
Kalaitzi et al. (2007)	Patients	Psychosexual intervention (one session/2 weeks for 10 weeks, for a total of five sessions)	No intervention	Couple	40 (20;20)	51.8
Rahmani et al. (2014)	Patients	Mindfulness-based stress reduction treatment or metacognition group therapy (2 h/a week for 8 weeks, for a total of eight sessions)	No intervention	Groups	24 (12;12)	43.25;44.92
Jun et al. (2011)	Survivors	Sexual life reframing program focused on the physical, psychological, and relational aspects of sexual health elements (2 h/a week per 6 weeks, for a total of six sessions)	Usual care	Groups	45 (22;23)	45.7
Sebastián et al. (2008)	Patients	Psychosocial intervention program (2 h/a week per 14 weeks, for a total of 14 sessions)	No intervention	Groups	175 (102;73)	48
Crane-Okada et al. (2012)	Patients	Cognitive: mindfulness and dance/movement activities (2 h/a week per 12 weeks, for a total of 12 sessions)	No intervention	Groups	41 (25;16)	65.6
Sherman et al. (2018)	Patients	Web-based psychological intervention (a single session)	Expressive writing	Groups	274 (132;142)	57.5

Outcome 1 includes six studies and nine dependent effect sizes on personal satisfaction with BI when naked or when dressed, BI distress, and BI appreciation. Outcome 2 includes four studies and eight dependent effect sizes related to sexual functioning, feeling attractive, sexual desire frequency, sexual interest, sexual dysfunction, and sexual satisfaction.

Details of each study included in the meta-analysis as well as descriptions of the effect sizes are provided in Tables 4, 5.

**Table 5:** Effect sizes of the studies included in the meta-analysis.

**Study**	**Dependent variable**	**Measure**	**Outcome**	***g***	**95%CI**	**Variance**	***z***
Svensk et al. (2009)	Body image	QLQ-BR23	1	0.36	−0.26; 0.98	0.1	−0.418
Svensk et al. (2009)	Sexual functioning	QLQ-BR23	2	0.23	−0.36; 0.82	0.09	−0.120
Kalaitzi et al. (2007)	Body image satisfaction when naked	*Ad hoc* questionnaire	1	1.41	0.73; 2.09	0.12	1.827
Kalaitzi et al. (2007)	Body image satisfaction when dressed	*Ad hoc* questionnaire	1	0.89	0.24; 1.54	0.11	0.715
Kalaitzi et al. (2007)	Feeling attractive	*Ad hoc* questionnaire	2	1.51	0.83; 2.19	0.12	1.797
Kalaitzi et al. (2007)	Sexual desire frequency	*Ad hoc* questionnaire	2	0.12	−0.50; 0.74	0.1	−0.285
Rahmani et al. (2014)	Body image (body cognition vs control)	QLQ-BR23	1	0.66	−0.12; 1.44	0.16	0.223
Rahmani et al. (2014)	Body image (mindfulness vs control)	QLQ-BR23	1	0.72	−0.09; 1.53	0.17	0.351
Rahmani et al. (2014)	Sexual functioning (body cognition vs control)	QLQ-BR23	2	−0.84	−1.65; −0.03	0.17	−1.722
Rahmani et al. (2014)	Sexual functioning (mindfulness vs control)	QLQ-BR23	2	0.30	−0.48; 1.08	0.16	−0.015
Jun et al. (2011)	Body image	CARES	1	−0.11	−0.70; 0.48	0.09	−1.423
Jun et al. (2011)	Sexual interest	CARES	2	0.20	−0.39; 0.79	0.09	−0.165
Jun et al. (2011)	Sexual dysfunction	CARES	2	0.13	−0.46; 0.72	0.09	−0.270
Jun et al. (2011)	Sexual satisfaction	CARES	2	0.83	0.24; 1.42	0.09	0.779
Sebastián et al. (2008)	Body image	BIS	1	0.77	0.49; 1.05	0.02	0.459
Sherman et al. (2018)	Body image distress	BID	1	0.07	−0.13; 0.27	0.01	−1.038
Sherman et al. (2018)	Body appreciation	BCSs	1	0.23	0.03; 0.43	0.01	−0.696

*Measures: QLQ-BR23, Breast Cancer-Specific Quality of Life Questionnaire (Sprangers et al., 1996; EORTC, 2010); CARES, Cancer Rehabilitation Evaluation System questionnaire (Ganz et al., 1992); BIS, Body Image Scale (Hopwood et al., 2001); BID, Body Image Distress (Galiano-Castillo et al., 2014; Paterson et al., 2015); BCS, body appreciation scale (Avalos et al., 2005)*.

*Outcome: 1 = body image; 2 = sexual functioning*.

### Assessment of Study Quality

The assessment of study quality revealed that most studies had an adequate quality (5 or more points out of 10 points). The details of the assessment of study quality included in this meta-analysis are reported in Table 6.

**Table 6:** Assessment of the quality of the studies.

**Study**	**Breast cancer patients or breast cancer survivors**	**Detailed description of the psychological intervention**	**Training conducted by a trained psychologist**	**Supervision during treatment or protocol screening for adherence**	**Intention to treat analysis**	**Randomization by a third party**	**Blind conditions**	**Control group without treatment**	**Absence of differential attritions between groups**	**Absence of reporting bias**	**Total**
Svensk et al. (2009)	Yes	Yes	Yes	No	No	Yes	Yes	Yes	Yes	Yes	8/10
Kalaitzi et al. (2007)	Yes	Yes	Yes	No	Yes	No	Yes	Yes	Yes	Yes	8/10
Rahmani et al. (2014)	Yes	Yes	N/A	No	Yes	No	Yes	Yes	Yes	Yes	7/10
Jun et al. (2011)	Yes	Yes	Yes	No	Yes	Yes	Yes	Yes^*^	Yes	Yes	9/10
Sebastián et al. (2008)	Yes	Yes	N/A	No	Yes	No	Yes	Yes	No	Yes	6/10
Crane-Okada et al. (2012)	Yes	Yes	Yes	No	Yes	Yes	Yes	Yes	No	Yes	8/10
Sherman et al. (2018)	Yes	Yes	N/A	No	No	Yes	Yes	No	No	Yes	5/10

*N/A, not available*.

* unspecified “usual care.”

### Risk of Bias

The quality appraisal of the selected studies was assessed through the Cochrane risk of bias tool, version 2 (RoB 2; Higgins et al., 2011). The researchers reached an overall consensus on the quality appraisal evaluation of the seven selected studies and the results are reported in Table 7. Only one (Sebastián et al., 2008) of the included studies had high risk of bias in sequence generation. Randomization methods of the other studies were clear, even if they did not explain how participants were allocated. About the blinding of participants and personnel, only Sherman et al. (2018) specified it. Sebastián et al. (2008) claimed the blinding of outcome data, while in the other studies it is unclear. Three of seven studies (Sebastián et al., 2008; Crane-Okada et al., 2012; Sherman et al., 2018) reported attrition bias because they had a dropout rate. No selective reporting bias was detected in included studies. Finally, three of seven studies (Kalaitzi et al., 2007; Svensk et al., 2009; Rahmani et al., 2014) reported other biases in their experimental studies. Other studies are unclear or the risk of bias was assessed as low.

**Table 7:** Risk of bias assessment.

	**Random sequence generation**	**Allocation concealment**	**Blinding of participants and personnel**	**Blinding of outcome data**	**Incomplete outcome data**	**Selective reporting**	**Other bias**
Svensk et al. (2009)	+	?	–	?	+	+	–
Kalaitzi et al. (2007)	+	?	–	?	+	+	–
Rahmani et al. (2014)	+	?	–	?	+	+	–
Jun et al. (2011)	+	?	–	?	+	+	?
Sebastián et al. (2008)	–	–	–	–	–	+	+
Crane-Okada et al. (2012)	+	?	–	?	–	+	?
Sherman et al. (2018)	+	?	+	?	–	+	?

*“+”, low risk of bias; “?”, unclear risk of bias; “–”, High-risk of bias*.

### Outlier Analysis

The outlier analysis conducted on all the dependent effect sizes included in this meta-analysis highlighted that standardized *z* values not exceeded ±3.29 (Table 5). Thus, all the effect sizes were included in the analyses.

### Outcome 1: The Efficacy of Psychological Intervention on Breast Cancer Patients' and Survivors' Body Image

**Figure 2:**
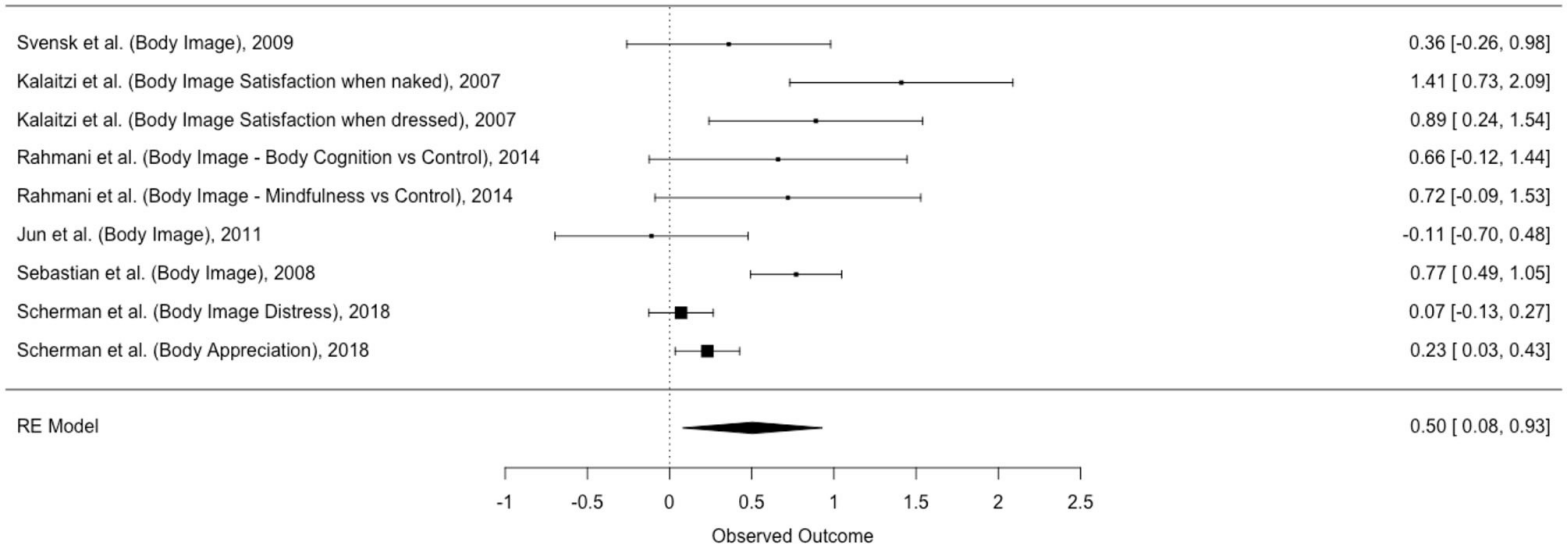
Forest plot of effect sizes of Body Image.

The first meta-analysis explored the efficacy of psychological intervention on breast cancer patients' and survivors' *Body Image* (Table 8). Six studies and nine non-independent effect sizes were included in the analysis (Figure 2).

**Table 8:** Results for the overall mean effect sizes of the two outcomes (body image and sexual functioning).

**Domain**	**# Studies/# ES**	**Mean *g* (SE)**	**95% CI**	***p***	**% Var. at level 1**	**Level 2 variance**	**% Var. at level 2**	**Level 3 variance**	**% Var. at level 3**
Body image	6/9	0.50 (0.18)	(0.08;0.93)	0.03	19.11	0.002	1.01	0.149	79.88
Sexual functioning related to body image	4/8	0.33 (0.22)	−0.20;0.85	0.19	27.37	0.286	72.63	0.000	0.00

*#Studies/#ES, Number of studies/ number of effect sizes; % Var. percentage of variance explained; Level 2 variance, variance between effect sizes from the same study; Level 3 variance, variance between studies*.

The random effect three-level analysis showed a medium, statistically significant effect of psychological interventions on breast cancer patients' and survivors' BI [*g* = 0.50; 95% CI (0.08; 0.93); *p* < 0.05].

**Figure 3:**
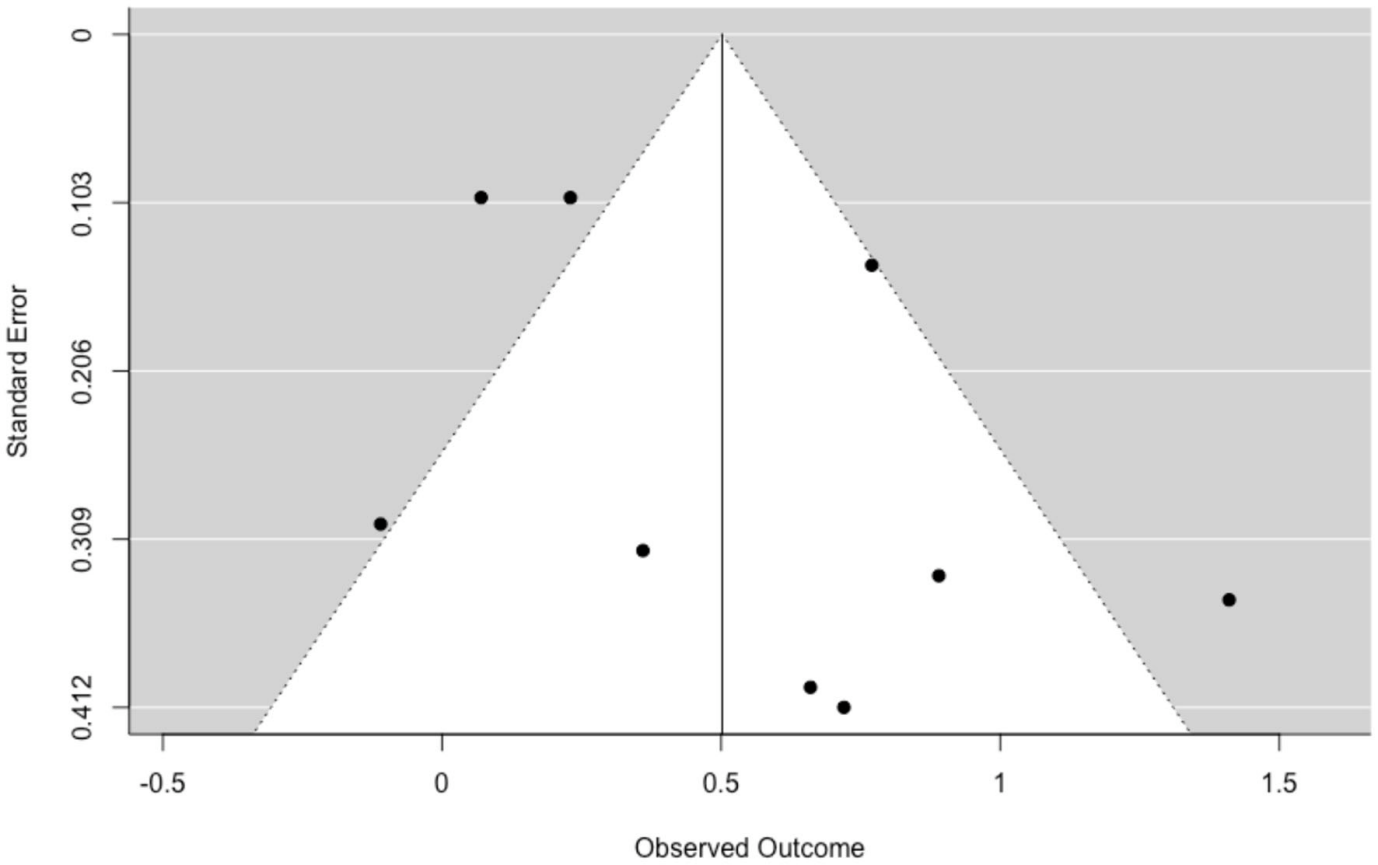
Funnel plot of publication bias in Body Image.

The funnel plot with trim and fill function shows no visual asymmetry and suggests a low probability of publication bias (Figure 3).

The heterogeneity was significant [*Q* (8) = 33.75, *p* < 0.001, *I*^2^ = 80.89]. The 19.11% of the total variance can be attributed to the sampling variance, 1.01% can be attributed to within studies variance (the differences between effect sizes within studies, and 79.88% can be attributed to between-studies variance (differences between studies). Log-likelihood tests revealed a non-statistically significant variance within-study (*p* = 0.89), and between-study (*p* = 0.07). The prediction interval is within −0.49 to 1.50.

### Outcome 2: The Efficacy of Psychological Intervention on Breast Cancer Patients' and Survivors' Sexual Functioning Related to Body Image

**Figure 4:**
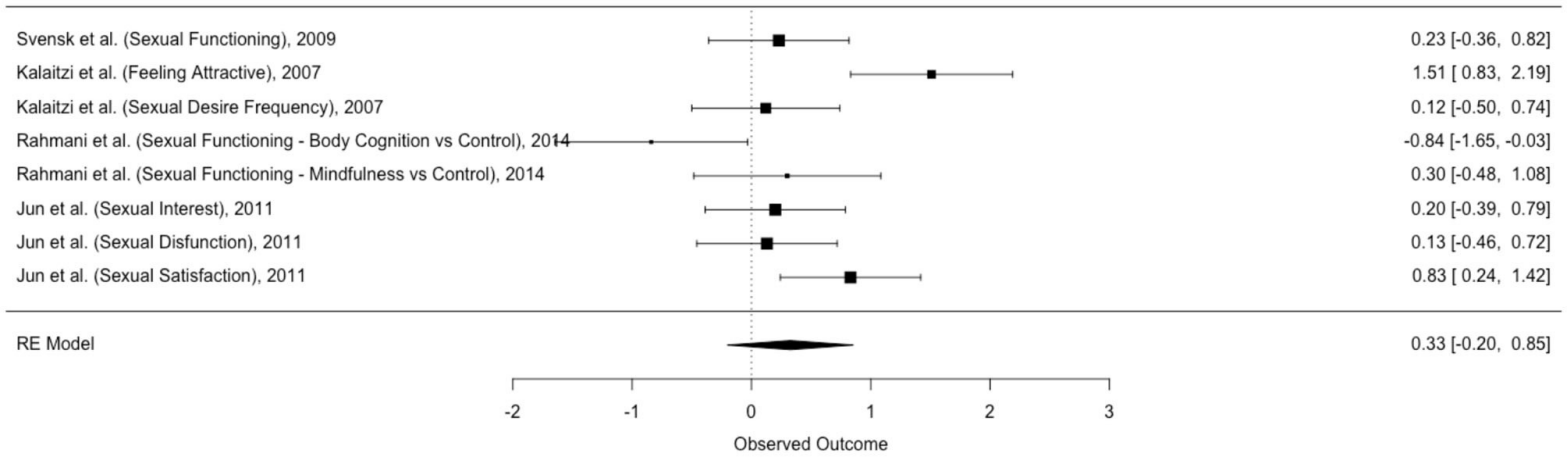
Forest plot of effect sizes of *Sexual Functioning Related to Body Image*.

The second meta-analysis explored the efficacy of psychological intervention on breast cancer patients' and survivors' *Sexual Functioning Related to Body Image* (Table 8). The dataset was composed by four studies and eight dependent effect sizes (Figure 4).

The three-level meta-analysis showed a non-statistical significant effect size {*g* = 0.33 [95% CI (−0.20; 0.85); *p* = 0.19]}, suggesting that psychological interventions do not have an impact on breast cancer patients and survivors' *Sexual Functioning Related to Body Image*. Interestingly, one study highlighted a negative effect size in sexual functioning (*g* = −0.84) (Rahmani et al., 2014).

**Figure 5:**
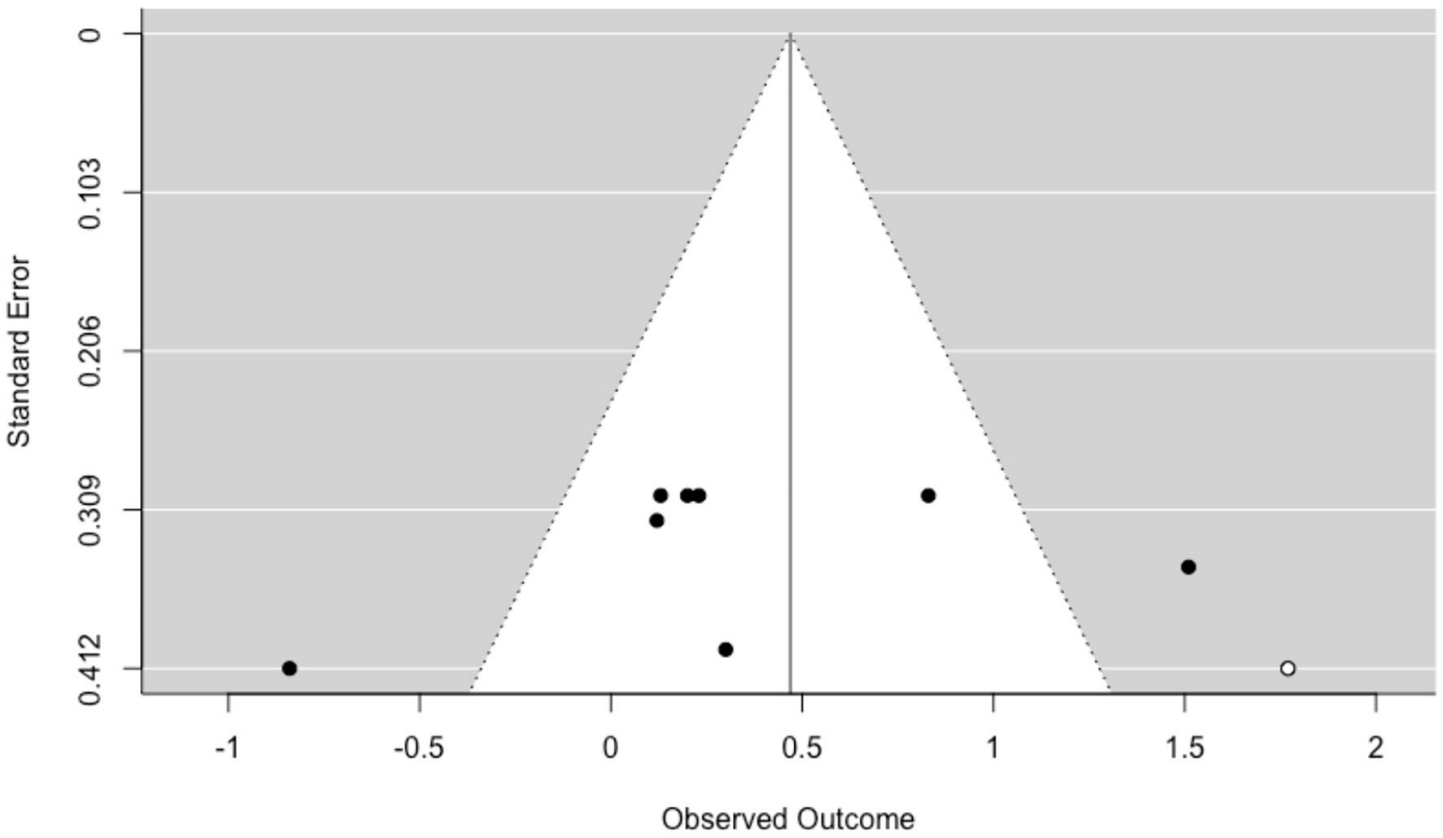
Funnel plot of publication bias in *Sexual Functioning related to Body Image*.

The funnel plot with trim and fill method (Figure 5) reveals only one missing effect size in the (bottom) right side of the graph, suggesting a low probability of publication bias.

The heterogeneity was significant [*Q* (7) = 23.60, *p* < 0.01, *I*^2^ = 72.63]. The 27.37% of the total variance can be attributed to the sampling variance, 72.63% can be attributed to within-studies variance, and 0% can be attributed to between-studies variance. Log-likelihood tests revealed a statistically significant variance within-study (*p* = 0.02), and a no-statistically significant variance between-study (*p* = 1.00). The prediction interval ranged between a large negative and a large positive effect size (−1.04 to 1.69).

## Discussion

The current study reviewed the literature prior to March 2020 about the efficacy of psychological interventions for BI and sexual functioning related to BI in breast cancer patients and survivors using meta-analytic techniques. BI is a crucial component of the experience of cancer, and especially of breast cancer. Indeed, patients' and survivors' bodies are not only affected by the disease but also by the secondary consequences of treatment such as surgery and chemotherapy (Munzone et al., 2019; Oliveri et al., 2020). Psycho-oncologists are well aware that alterations of BI go way beyond a mere “self-appreciation” issue: the body is felt as less whole, less feminine, and as a source of danger and betrayal (Rubin and Tanenbaum, 2011; Triberti et al., 2019a). Women are directly affected by this perception in their intimacy and sexual life, as well as in their perceived ability to give birth and nurture (Markopoulos et al., 2009; Faccio et al., 2020). Since body image affects patients and survivors' quality of life strongly, relevant changes in BI impact on psychological well-being in terms, for example, of self-esteem and social life. Identifying psychological interventions which impact on patients' and survivors' BI is relevant to sustain the adjustment to illness, especially to chronic illness as breast cancer. Findings suggested that different types of psychological interventions are effective for breast cancer patients' and survivors' BI with a medium effect size. This suggests that the improvements are not modality-specific but there are various explanations for the benefits in the studies reviewed. However, in this meta-analysis, cognitive, social, and sexual interventions as well as art therapy, mindfulness, and web-based treatments are assessed as types of psychological interventions. In addition, except for the single session in the web-based psychological treatment by Sherman et al. (2018), psychological interventions are extended and consistent over time (from 5 to 14 weeks for each intervention). Moreover, in these studies professionals delivering psychological interventions are psychologists and draw on their training to propose mixed methods to engage many aspects of BI linked to psychological, emotional, and social changes. This may enable breast cancer patients and survivors to address various aspects of BI without self-fragmentation. Although BI received attention in the psycho-oncology literature in the last few decades, a first issue regarding BI conceptualization in breast cancer patients and survivors. Given the complexity of BI definition as a multidimensional construct, positive outcomes on BI do not depend on specific characteristics of psychological interventions but in meeting patients and survivors needs and preferences, as affirmed by Möller et al. (2019). Personalized interventions and collaborative psychological interventions (e.g., Smith and George, 2012; Aschieri et al., 2015; Durosini et al., 2017) are needed to deal with BI issues taking into account any individual breast cancer patient's or survivor's situation and experiences. Referring to sexual functioning, literature reveals the association between BI and sexual functioning in breast cancer patients and survivors (Woertman and Van den Brink, 2012). BI issues interfere with sexual functioning due to the connection between how a woman perceives her own body and sexuality (Seal, Andrea and Cindy). This could be particularly relevant for breast cancer patients and survivors who have to cope with an ill body during and/or after oncological treatments. However, in this meta-analysis sexual functioning does not show improvement among the reviewed contributions. One possible explanation might be related to the typology of psychological interventions in which sexual functioning is not the main objective of interest. Moreover, some studies, such as the art therapy program by Svensk et al. (2009) in the field of cognitive-based interventions and the study of Rahmani et al. (2014), do not focus on sexual issues specifically. Without a specific focus on sexuality, it is possible that this contribution's intervention was not effective in this regard.

In addition, it has been suggested that BI is extensively entwined with social ideals in a particular place and time (Perdue et al., 2018). The comparison between the actual and ideal appearance can increase the self-objectification that is strongly connected to disease in sexual functioning (Bishop, 2015). This is particularly evident in breast cancer patients and survivors because tumor experience affects cognitions and perceptions about BI with direct influences on, for example, perception of being attractive and sexual desire (Benoit, 2020). Relevant changes in the body can be both visible (e.g., scarring and hair loss) and not apparent (e.g., interoception or the inner perception of the body). This suggests that sexual functioning has to be assessed as one of the main aspects of interest related to BI, especially in breast cancer patients and survivors. Finally, the low number of available studies focused on BI issues shows that BI has not yet been explored enough in the current literature and sustains the need to promote psychological interventions in this field.

## Conclusion

Drawing researchers' attention to the multiple characteristics associated with BI, this study aimed to explore the efficacy of psychological interventions by involving both individual and relational aspects of BI. Findings show the positive outcomes on BI, thanks to both individual and group psychological interventions conducted by trained professionals. The usage of strict criteria for data selection and cultural differences could be limitations of this meta-analysis. Additionally, keywords related to specific types of psychological interventions were not used as well as gray literature and doctoral dissertation were excluded from the selected studies. Thus, it is possible that some studies were not identified as fulfilling the selection criteria adequately and the literature search might be not sufficient to provide a comprehensive and full picture of the evidence. In the same line, only three databases were used for the study research without relevant medical databases related to breast cancer issues. Furthermore, the authors suggest a cautious evaluation of the funnel plot and the trim-and-fill method due to their low sensitivity to detect publication bias when the number of studies is small as in this meta-analysis. At the same time, the high statistical variance across study designs, as result of different levels of clinical and methodological diversity (e.g., the inclusion of a non-randomized trial), is a limitation of the study.

Regarding future directions, research may feature more psychological characteristics, improve measurements, and change inclusion criteria of study selection. Additionally, it may be interesting to explore the effectiveness of psychological interventions on cancer patients and survivors moderated by the type of psychological intervention and the typology of participants. Third, qualitative studies could be reviewed to assess the individual perceptions of patients and survivors with the aim to explore BI dimensions and implement personalized psychological interventions centered on the lived illness. Recommendation for clinical practice suggests that psychological interventions have to assess BI and related constructs in terms of BI appreciation as well as sexual functioning. Therefore, these findings highlight the foundation for individualized aids in meeting breast cancer patients' and survivors' needs, given more space to sexual functioning and their issues linked to cognitive and social processes.

## Data Availability Statement

The raw data supporting the conclusions of this article will be made available by the authors, without undue reservation.

## Author Contributions

VS and ID conceived the ideas presented in the article and wrote the first draft. VS, ID, and ST performed the literature search. ID performed the meta-analysis. ST contributed to the interpretation of the data, provided comments on the ideas presented, and edited the manuscript. GP contributed with important intellectual contents and supervised the whole process. All listed authors gave final approval of the version to be published.

## Conflict of Interest

The authors declare that the research was conducted in the absence of any commercial or financial relationships that could be construed as a potential conflict of interest.
